# Recent advanced lipid-based nanomedicines for overcoming cancer resistance

**DOI:** 10.20517/cdr.2024.19

**Published:** 2024-06-21

**Authors:** Piroonrat Dechbumroong, Runjing Hu, Wisawat Keaswejjareansuk, Katawut Namdee, Xing-Jie Liang

**Affiliations:** ^1^CAS Key Laboratory for Biomedical Effects of Nanomaterials and Nanosafety, CAS Center for Excellence in Nanoscience, National Center for Nanoscience and Technology of China, Beijing 100049, China.; ^2^School of Nanoscience and Engineering, University of Chinese Academy of Sciences, Beijing 100049, China.; ^3^National Nanotechnology Center (NANOTEC), National Science and Technology Development Agency, Pathum Thani 12120, Thailand.; ^#^Authors contributed equally.

**Keywords:** Cancer resistance, lipid-based nanomaterials, nanomedicines, drug delivery

## Abstract

The increasing prevalence of cancer drug resistance not only critically limits the efficiency of traditional therapies but also causes relapses or recurrences of cancer. Consequently, there remains an urgent need to address the intricate landscape of drug resistance beyond traditional cancer therapies. Recently, nanotechnology has played an important role in the field of various drug delivery systems for the treatment of cancer, especially therapy-resistant cancer. Among advanced nanomedicine technologies, lipid-based nanomaterials have emerged as effective drug carriers for cancer treatment, significantly improving therapeutic effects. Due to their biocompatibility, simplicity of preparation, and potential for functionalization, lipid-based nanomaterials are considered powerful competitors for resistant cancer. In this review, an overview of lipid-based nanomaterials for addressing cancer resistance is discussed. We summarize the recent progress in overcoming drug resistance in cancer by these lipid-based nanomaterials, and highlight their potential in future applications to reverse cancer resistance.

## INTRODUCTION

Cancer is one of the foremost causes of mortality globally. In 2023, the American Cancer Society estimated the number of new cancer cases as high as 1,958,310 in the U.S. and 609,820 cases would be accounted by cancer-related fatality, approximating 4 new cases and 1 death every minute^[1]^. Nowadays, combinations of surgery, radiation therapy, and chemotherapy are the standard regimen for cancer management. However, the success of the therapeutic approaches is known to be affected by off-target effects and severe drug resistance in many types of cancer such as breast cancer^[2,3]^, lung cancer^[4]^, colon cancer^[5]^, and prostate cancer^[6,7]^.

Drug resistance that the cancer cells withstand a wide range of treatments is a crucial problem in cancer management, and is associated with relapses or recurrences, significantly hindering the cure^[8-12]^. Based on the timing of drug resistance development, it can be divided into intrinsic resistance and acquired resistance. Intrinsic resistance exists before the beginning of therapy, which is possibly caused by tumor heterogeneity subpopulations, inherent genetic mutations, and activation of intrinsic detoxification pathways. Conversely, acquired resistance occurs after therapy, which results from post-treatment changes in tumor microenvironment (TME), activation of second proto-oncogenes or tumor suppressor genes, and mutations or alteration of drug target expression levels after treatment. Additionally, drug resistance can be clinically categorized based on specific mechanisms including tumor heterogeneity, increased efflux of drugs, enhanced DNA damage repair, altered drug target, epithelial-mesenchymal transition, and so on^[12-21]^. Theoretically, retaining cytotoxic agents within the cells, erasing reversible modifications, modifying the TME, and delivering combinations of cytotoxic drugs with different mechanisms to cancer cells are basic principles for circumventing cancer drug resistance. These approaches may re-sensitize the patients to the treatment^[22,23]^.

**Figure 1:**
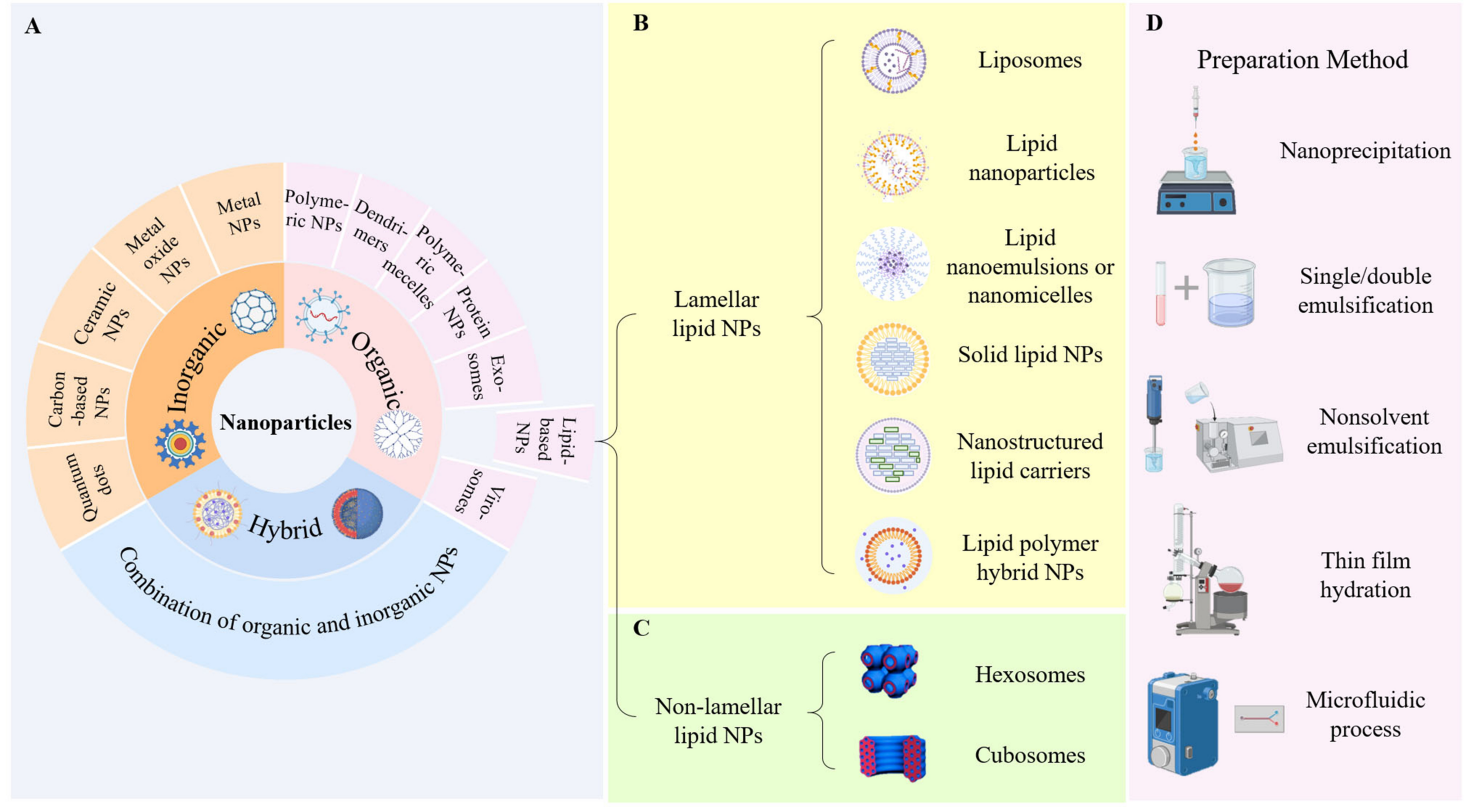
Schematic presentation. (A) classification of nanomaterials; (B) classification of lipid-based nanomaterials: lamellar NPs include liposomes, LNPs, LNEs, SLNs, NLCs, and lipid polymer hybrid NPs; (C) non-lamellar NPs include hexosomes and cubosomes; (D) typical preparation methods of lipid-based nanomaterials. (A, B and D) are created by www.BioRender.com; (C) is quoted with permission from Tan *et al.*^[29]^. NPs: Nanoparticles; LNPs: lipid nanoparticles; LNEs: lipid nanoemulsions; SLNs: solid lipid nanoparticles; NLCs: nanostructured lipid carriers.

To date, studies on treatment strategies to overcome drug resistance have been conducted for a better understanding of different resistance mechanisms. Nanotechnology-based therapy is typically referred to as medical treatments that utilize nanoscale materials and devices to diagnose, monitor, and treat disease. The products developed from this technology are referred to as nanomedicines and nanoformulations, which are materials systems consisting of appropriate nanocarriers and active pharmaceutical ingredients^[24,25]^. Consequently, the effective functioning of nanomedicines relies on the utilization of nanoparticles. Typically, nanoparticles are classified into three categories based on their compositions: (1) inorganic nanoparticles; (2) organic nanoparticles; and (3) hybrid nanoparticles [Figure 1A]^[26-29]^. Numbers of studies have demonstrated the promising results of nanomaterials in nanoformulations to enhance efficiency and minimize the severe off-target effects^[30-35]^. The potential of nanotechnology-based therapy in anti-drug resistance strategies is attracting significant attention^[23,36-39]^.

Nanoformulations are defined by the U.S. Food and Drug Administration (FDA) as products in combination with nanoparticles having one or more dimensions ranging from 1-100 nanometers (nm) or up to 1,000 nm if the size is engineered to show specific dimension-dependent properties or phenomena^[40-43]^. However, there is no universally recognized definition for nanoformulations^[44]^. The typical size preferred in nanomedicine applications is in the range of 100-200 nm due to its ability to cross microcapillaries and capacity to accommodate an adequate amount of active pharmaceutical agents^[45]^. Doxil®, the first nanoformulation of doxorubicin (DOX) encapsulated in 100 nm liposomes, was approved in 1995. It remains a first-line cancer treatment to the present. The lower cardiotoxicity and enhanced efficiency in killing tumor cells of Doxil® increase chemotherapy sensitivity and diminish the drug resistance over the free drug molecule^[46]^. The excellent therapeutic effects demonstrated by nanoformulations compared to free drug molecules, including improved pharmacokinetics, enhanced target specificity, and reduced systemic toxicity, have garnered significant interest in the development of anticancer drugs^[47]^. Subsequently, robust nanomedicines have been continuously developed and approved for cancer treatment [Table 1]. It is worth noting that among nanoformulations launched on the market for clinical cancer treatment, lipid-based nanomaterials are the frontrunners^[48]^.

**Table 1:** List of approved nanomedicines for cancer treatment^[23,26,47-59]^

**No.**	**Description of carrier and its compositions**	**Product names**	**Manufacturer**	**Indication(s)**	**Approval agency (year)**
**Liposomes**					
1	PEGylated liposomal doxorubicin (MPEG-DSPE, HSPC, cholesterol)	Doxil®, Caelyx®	Janssen	AIDS-related Karposi sarcoma, ovarian cancer, multiple myeloma	FDA (1995, 2005, 2008) EMA (1996)
2	Non-PEGylated liposomal daunorubicin (DSPC, cholesterol)	DaunoXome®	Galen	Advanced AIDS-related Kaposi’s sarcoma	FDA (1996)
3	Non-PEGylated liposomal cytarabine (DOPC, DPPG, cholesterol, triolein)	DepoCyt®	DepoTech Corporation	Intrathecal treatment of lymphomatous meningitis	FDA (1999)
4	Non-PEGylated liposome doxorubicin (PC, cholesterol)	Myocet®	Teva B.V.	Metastatic breast cancer	EMA (2000)
5	Non-PEGylated liposome paclitaxel	Lipusu®	Luye Pgarna	Breast cancer, NSCLC, lung squamous cell carcinoma	NPMA, P.R. China (2003)
6	Non-PEGylated liposomal mifamurtide (POPC, OOPS)	Mepact®	Takeda France SAS	Non-metastatic osteosarcoma	EMA (2009)
7	Non-PEGylated liposomal vincristine (SPH, cholesterol)	Marqibo®	Talon Therapeutics	Philadelphia chromosome-negative acute lymphoblastic leukemia (tertiary)	FDA (2012)
8	PEGylated liposome doxorubicin (HSPC, DSPE-PEG, cholesterol)	Lipodox®	Sun Pharmaceutical Industries Ltd.	AIDS-associated Kaposi’s sarcoma, metastatic ovarian cancer, multiple myeloma	FDA (2013)
9	PEGylated liposomal irinotecan (DSPC, MPEG-2000-DSPE, cholesterol)	Onivyde®	Merrimack	Advanced pancreatic cancer	FDA (2015)
10	Non-PEGylated liposome cytarabine:daunorubicin (5:1 M ratio) (DSPC, DSPG, cholesterol)	Vyxeos® (CPX-351)	Jazz Pharmaceuticals	Acute myeloid leukemia	FDA (2017) EMA (2018)
11	PEGylated liposome doxorubicin (MPEG-2000-DSPE, HSPC, cholesterol)	Zolsketil®	Accord Healthcare S.L.U.	AIDS-associated Kaposi’s sarcoma, metastatic breast cancer, advanced ovarian cancer, multiple myeloma	EMA (2022)
**Nanomicelles**					
12	Docetaxel (Polysorbate 80)	Taxotere®	Sanofi-Aventis	Advance/metastasis breast cancer	FDA (1996)
13	Paclitaxel (NIPAM, VP)	Nanoxel®	Samyang Biopharmaceuticals	Metastasis breast cancer, NSCLC, ovarian cancer	MFDS (2012)
14	Paclitaxel (N-(all-trans-retinoyl)-L-cysteic acid methyl ester sodium salt, N-(13-cis-retinoyl)-L-cysteic acid methyl ester sodium salt)	Paclical®	Oasmia Pharmaceuticals	Ovarian cancer	RFMPH (2015)
15	Micelles paclitaxel (N-(all-trans-retinoyl)-L-cysteic acid methyl ester sodium salt, N-(13-cis-retinoyl)-L-cysteic acid methyl ester sodium salt)	Apealea®	Inceptua AB	Ovarian cancer, peritoneal cancer, fallopian tube cancer	EMA (2018, withdrawn in 2024)
**Protein-bound nanoparticles**					
16	Engineered protein combining IL-2 and diphtheria toxin	Ontak®	Eisai	Persistent or recurrent cutaneous T-cell lymphoma	FDA (1999)
17	Human albumin-bound paclitaxel NP	Abraxane® (ABI-007)	Celgene	Metastatic breast cancer, NSCLC, metastatic pancreatic cancer	FDA (2005, 2012, 2013) EMA (2008)
18	Antibody-drug conjugate (trastuzumab, DM1)	Kadcyla®	Roche Registration GmbH	Early breast cancer, metastatic breast cancer	FDA (2013) EMA (2013)
19	Human albumin-bound paclitaxel NP	Pazenir®	Ratiopharm GmbH	Metastatic breast cancer, NSCLC	EMA (2019)
**Polymer nanoparticles: synthetic polymer particles**					
20	Polymer-protein conjugate PEGylated L-asparaginase (MPEG)	Oncaspar®	Enzon Pharmaceuticals	ALL and hypersensitivity to asparaginase	FDA (1994, 2006)
21	Leuprolide acetate and polymer (PLGH or PLG)	Eligard®	Tolmar	Advanced prostate cancer	FDA (2002)
22	PEG-PLA polymeric micelle paclitaxel	Genexol-PM®, Cynviloq®	Samyang Biopharmaceuticals	Metastasis breast cancer, NSCLC, ovarian cancer	MFDS (2007)
23	Nanodispersion of a taxane derivative; PICN	Bevetex®	Sun Pharma Advanced Research Company Limited	Metastatic breast cancer	India (N/A)

PEG: Polyethylene glycol; MPEG-DSPE: N-(carbonyl-methoxypolyethylene glycol 2000)-1,2-distearoyl-sn-glycero-3-phosphoethanolamine sodium salt; HSPC: fully hydrogenated soy phosphatidylcholine; FDA: Food and Drug Administration; EMA: European Medicines Agency; DSPC: 1,2-distearoyl-sn-glycero-3-phosphocholine or distearoylphosphatidylcholine; DOPC: 1,2-dioleoyl-sn-glycero-3-phosphocholine; DPPG: 1,2-dipalmitoyl-sn-glycero-3-phosphoglycerol; PC: phosphatidylcholine; NSCLC: non-small cell lung cancer; NPMA: National Medical Products Administration of the P.R. China; POPC: palmitoyl-2-oleoyl-sn-glycero-3-phosphocholine; OOPS: 1,2-dioleoyl-sn-glycero-3-phospho-L-serine monosodium salt; SPH: sphingomyelin; DSPE-PEG: sodium methoxy PEG 40-carbonyl-distearoylphosphatidylethanolamine; MPEG-2000-DSPE: N-(carbonyl-methoxypolyethyleneglycol-2000)-1,2-distearoly-sn-glycero-3-hosphoethanolamine; DSPG: 1,2-distearoyl-sn-glycero-3-phospho-(1’-rac-glycerol); NIPAM: N-isopropyl acrylamide; VP: vinyl pyrrolidone; MFDS: The Korean Ministry of Food and Drug Safety; RFMPH: Russian Federation: Ministry of Public Health; IL-2: interleukin-2; NP: nanoparticle; DM1: mertansine; ALL: acute lymphocytic leukemia; MPEG: monomethoxypolyethylene glycol; PLGH or PLG: poly (D,L-lactide-coglycolide); PLA: polylactic acid; PICN: paclitaxel injection concentrate for nanodispersion.

**Figure 2:**
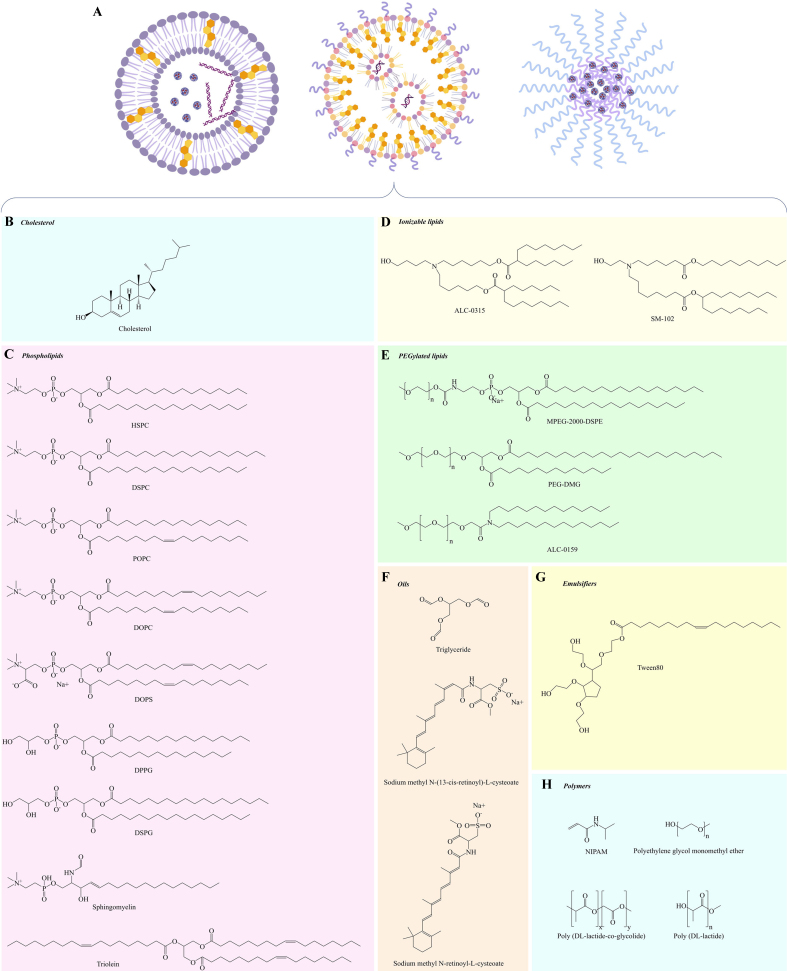
Molecular structures of common components used in lipid-based nanomedicines for cancer drug research. Group (A) representative lipid-based nanomaterials for cancer drug research include liposomes, LNPs, and nanomicelles (from left to right); group (B) cholesterol; group (C) phospholipids such as HSPC, DSPC, POPC, DOPC, DOPS, DPPG, DSPG, sphingomyelin, and triolein; group (D) ionizable lipids such as ALC-0315 and SM-102; group (E) PEGylated lipids such as MPEG-2000-DSPE, PEG-DMG, and ALC-0159; group (F) oils such as triglyceride, N-(13-cis-retinoyl)-L-cysteic acid methyl ester sodium salt, and N-(all-trans-retinoyl)-L-cysteic acid methyl ester sodium salt; group (G) emulsifiers such as Tween 80; and group (H) polymers such as NIPAM, polyethylene glycol monomethyl ether, poly (DL-lactide-co-glycolide), and poly (DL-lactide). (A) is created by www.Biorender.com, and all chemical structures are generated by ChemDraw Software. LNPs: Lipid nanoparticles; HSPC: fully hydrogenated soy phosphatidylcholine; DSPC: 1,2-distearoyl-sn-glycero-3-phosphocholine or distearoylphosphatidylcholine; POPC: palmitoyl-2-oleoyl-sn-glycero-3-phosphocholine; DOPC: 1,2-dioleoyl-sn-glycero-3-phosphocholine; DOPS: 1,2-dioleoyl-sn-glycero-3-phospho-l-serine; DPPG: 1,2-dipalmitoyl-sn-glycero-3-phosphoglycerol; DSPG: 1,2-distearoyl-sn-glycero-3-phospho-(1’-rac-glycerol); ALC-0315: (4-hydroxybutyl)azanediyl)bis(hexane-6,1-diyl)bis(2-hexyldecanoate); SM-102: (heptadecan-9-yl8-((2-hydroxyethyl)[6-oxo-6(undecyloxy)hexyl]amino)octanoate); PEG: polyethylene glycol; MPEG-2000-DSPE: N-(carbonyl-methoxypolyethyleneglycol-2000)-1,2-distearoly-sn-glycero-3-hosphoethanolamine; PEG-DMG: 1,2-dimyristoyl-rac-glycero-3-methoxypolyethylene glycol; ALC-0159: 2-[(polyethylene glycol)-2000]-N,N-ditetradecylacetamide; NIPAM: N-isopropyl acrylamide.

Lipid-based nanomaterials are nanoparticles whose structures are composed of lipids with good encapsulation potential, biocompatibility, and simplicity of preparation. They can be classified into two main types [Figure 1B and C]. First, lamellar lipid nanomaterials include liposomes, lipid nanoparticles (LNPs), lipid nanoemulsions (LNEs) or nanomicelles, solid lipid nanoparticles (SLNs), nanostructured lipid carriers (NLCs), and lipid polymer hybrid nanoparticles (LPHNPs)^[60]^. Second, non-lamellar lipid nanoparticles include nanostructured liquid crystalline particles such as cubosomes and hexosomes^[60-64]^. The compositions of lipid-based nanomaterials include cholesterol, phospholipids, polymers, and oils to form the structure of lipid-based nanomaterials; helper lipids and PEGylated lipids to enable long circulation inside the body and endosomal escape; and emulsifiers functioning as surfactants. Molecular structures of the lipid-based nanomaterials’ compositions and their limitations are shown in Figure 2 and Table 2. Nanoprecipitation, nonsolvent emulsification, thin film hydration, single/double emulsification, microfluidic mixing, and supercritical fluid techniques are typical synthesis methods [Figure 1D]^[61]^.

**Table 2:** Compositions of different lipid-based nanomaterials and their limitations^[60,65-70]^

**Types**	**Compositions**	**Advantages**	**Limitations**	**Status**
**Lamellar lipid nanomaterials**				
Liposomes	Phospholipids (e.g., PC, PS, PE, PG) and cholesterol	Simplicity of preparation Enhanced solubility Biocompatibility Biodegradability Non-immunogenicity Low toxicity	Drug leakage Short half‑life Low reproducibility Possible oxidation and hydrolysis	Commercial products and under clinical trials
LNPs	Phospholipids, cholesterol, helper lipids (e.g., DSPC, DPPC, DOPE) and PEGylated lipids (e.g., ALC-0159, DMG-PEG2000, DSPE-PEG2000)	High drug loading Long half-life Biocompatibility Biodegradability Non-immunogenicity Low toxicity	High cost Require specific storage conditions	Preclinical studies
LNEs or nanomicelles	Oils (e.g., triglycerides, vegetable oil) and surfactants (e.g., Tween 80)	Self-assembly High reproducibility High penetration to biological membranes	Cytotoxicity due to surfactant Possibility of phase separation	Commercial products and under clinical trials
SLNs	Solid lipids (triglycerides, fatty acids, waxes) and emulsifiers, surfactants or polymers	High stability Biocompatibility Free-organic solvents (green synthesis) Reproducibility Ease of scale-up process	Moderate encapsulation efficiency Crystallization Polymorphic transitions High drug expulsion Short shelf-life	Preclinical studies
NLCs	Solid lipids, liquid lipids (e.g., glyceryl tricaprylate, ethyl oleate, isopropyl myristate, and glyceryl dioleate) and emulsifiers	Increasing drug loading Low drug expulsion Improving permeability Increasing half-life	High operating tempurature Moderate drug loading Low stability	Preclinical studies
LPHNPs	Lipids and polymer (PCL, PLGA, PLA, PbAE, and chitosan)	Flexibility for surface modification High stability Long half-life Control released profile	High cost Difficult to scale up	Preclinical studies
**Non-lamellar lipid nanoparticles**				
Hexosomes (2D structure), cubosomes (3D structure)	Amphiphilic lipids (monoolein, diolein, phytantriol, phospholipids) and polymeric stabilizer	Highly curved membrane High surface area High loading efficiency (peptides and protein-based drugs)	Early stage of development	Preclinical studies

PC: Phosphatidylcholine; PS: phosphatidylserines; PE: phosphatidylethanolamines; PG: phosphatidylglycerols; LNPs: lipid nanoparticles; DSPC: 1,2-distearoyl-sn-glycero-3-phosphocholine or distearoylphosphatidylcholine; DPPC: 1,2-dipalmitoylphosphatidylcholine; DOPE: 1,2-dioleoyl-sn-glycero-3-phosphoethanolamine; PEG: polyethylene glycol; ALC-0159: 2-[(polyethylene glycol)-2000]-N,N-ditetradecylacetamide; DMG-PEG2000: 1,2-dimyristoyl-rac-glycero-3-methoxypolyethylene glycol-2000; DSPE-PEG: sodium methoxy PEG 40-carbonyl-distearoylphosphatidylethanolamine; LNEs: lipid nanoemulsions; SLNs: solid lipid nanoparticles; NLCs: nanostructured lipid carriers; LPHNPs: lipid polymer hybrid nanoparticles; PCL: polycaprolactone; PLGA: poly(lactic-co-glycolic acid); PLA: polylactic acid; PbAE: poly β-amino ester.

Besides good biocompatibility, lipid-based nanomedicines and their derivatives have shown promising results in addressing issues related to immunogenicity^[60,69]^. The flexibility of the cargo structure allows easy modifications to achieve desired properties. Surface functionalization, such as PEGylation and decoration with targeting ligands, including nucleic acid, peptides, aptamers, and antibodies, is one of the promising strategies to make lipid-based nanomaterials a powerful weapon against cancer drug resistance^[27,71-75]^. Furthermore, preparation methods, ingredient types and ratios, combination of biocompatible polymers, biomimetic engineering techniques, functional cancer cell-membranes integration, and physicochemical or biochemical trigger mechanisms are all utilized to design advanced lipid-based nanomaterials^[70,76]^. Combining these modification techniques can adjust lipid structural packing, characteristics, composition at the atomic level, morphology, encapsulation efficiency, biocompatibility, and delivery profile of lipid-based nanomaterials, resulting in improved therapeutic outcomes^[50,77]^. Following this, researchers and scientists are intensively studying multifunctional lipid-based nanomaterials mainly for the treatment of different cancer types.

Generally, delivering lipid-based nanomedicines to tumor sites is primarily achieved through passive targeting via the enhanced permeability and retention effect (EPR), which preferentially allows them to accumulate in tumors. Notably, nanocarriers can enter cells through three main routes: passive targeting, active targeting, and stimulus-sensitive structures. Firstly, passive targeting occurs when lipid-based nanomaterials penetrate the tumor cellular membranes based on their inherent physicochemical properties. Secondly, active targeting involves modifying the nanocarrier structures with specific moieties that selectively recognize and bind to tumor cells. Lastly, stimulus-sensitive structures regulate the delivery profile of anticancer drugs by responding to external or internal triggers such as pH, temperature, magnetic field, ray, ultrasound, and so on^[76,78,79]^.

**Figure 3:**
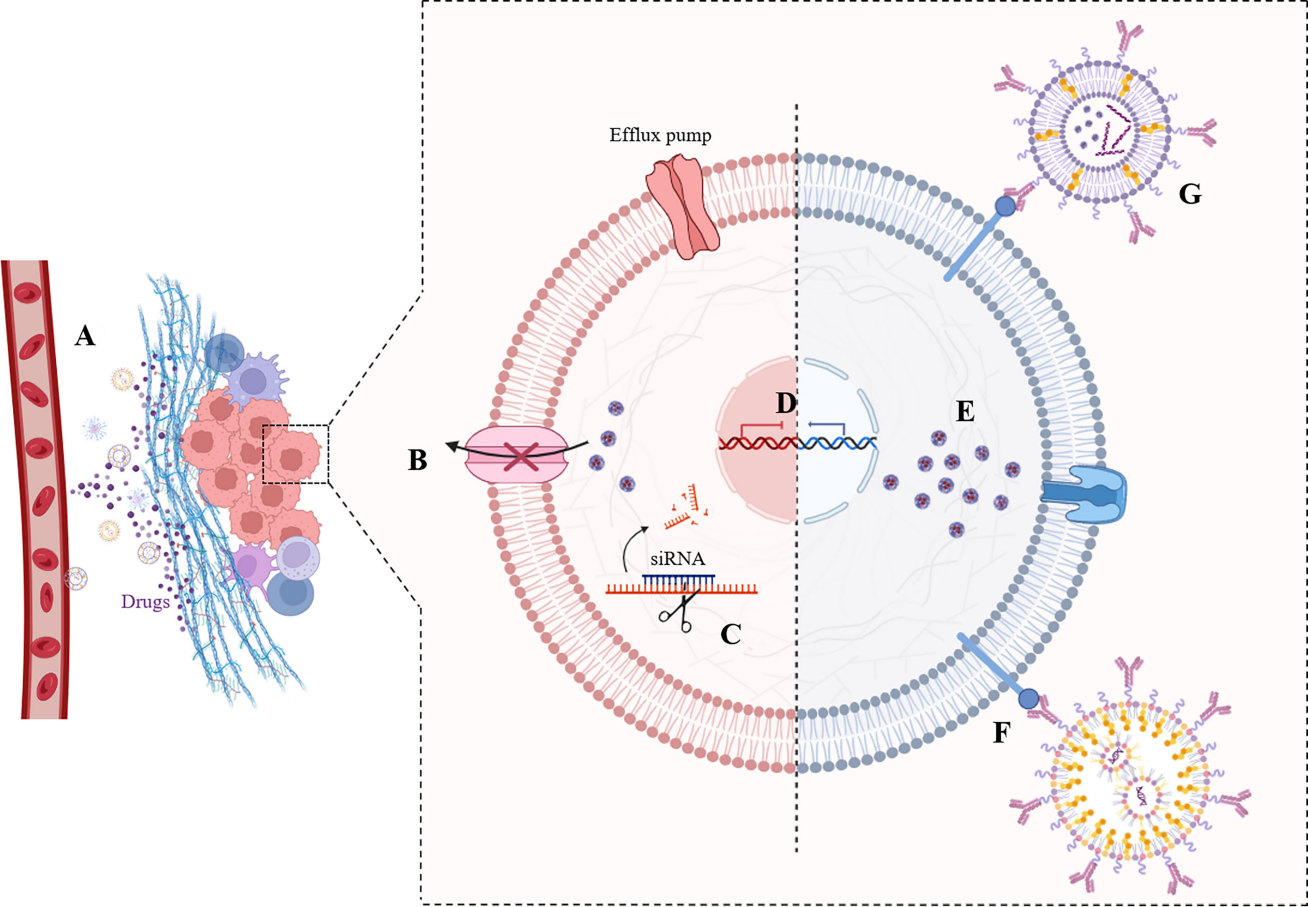
Illustration of the tactics employed in combatting drug-resistant cancer through the utilization of lipid-based nanomaterials. (A) remodeling of TME; (B) efflux pump inhibition; (C) knockdown or downregulation of anti-apoptotic protein expression pathway; (D) knockdown gene; (E) enhanced drug accumulation; (F) specific targeted drug delivery; and (G) co-delivery systems. Created by www.BioRender.com. TME: Tumor microenvironment.

Through internalization mechanisms and the customizations of lipid-based nanomaterials, anticancer agents with lipid-based nanomaterials are showing promise in overcoming drug resistance due to their small size, enabling tissue penetration and deep accumulation in tumor tissues, specific targeting, extension of drug circulation in the body, the ability to contain agents with different anticancer mechanisms in one carrier, inhibition of reversible modifications, and alteration of the TME [Figure 3]. Moreover, lipid-based nanomaterials are also capable of co-administration with other treatments to improve therapeutic outcomes and have gained interest as a strategy to cope with drug resistance. Providing a valuable platform for cancer treatment, lipid-based nanomaterials offer material flexibility of their own^[60,61]^.

In this review, the lipid-based nanocarriers including liposomes, LNPs, and nanomicelles are discussed due to their approved and long-standing usage in anticancer nanomedicines. The recent advances to overcome cancer drug resistance using lipid-based nanomedicines and related strategies are summarized. However, the mechanisms of cytotoxic agents are not discussed in this review.

## LIPID-BASED NANOMATERIALS FOR CANCER RESISTANCE

### Liposomes

Liposomes, classical nanocarriers based on lipid materials, were initially identified in 1965 by Bangham *et al.*^[80]^. Structurally, they are spontaneously self-assembling small sphere artificial closed vesicles with sizes varying from 20 to 1,000 nm^[36,60,81]^. Typical components of liposomes include phospholipids, such as phosphatidylcholines (PC), phosphatidylethanolamines (PE), phosphatidylglycerols (PG), and phosphatidylserines (PS), along with stabilizers such as cholesterol^[60]^. Nowadays, several nano-delivery systems have been incorporated into clinically used medicines for cancer treatment, including lipid-based nanoparticles, protein-bound nanoparticles, and polymer nanoparticles [Table 1]. Of those, liposomes are particularly promising platforms because of their loading capacity with different hydrophilicity, their superior membrane fusion abilities, and their ease of modifiable properties. In addition, the cell membrane-like structure and components enable liposomes to deliver drugs into cells through membrane fusion and endocytosis, thereby improving the accumulation of drugs in the cytoplasm^[78]^.

Various techniques, including PEGylation and specific ligand attachment, have been employed to enhance the stability and selectivity of liposomes *in vivo*, thus reducing the occurrence of drug resistance^[36,60,73,81]^. To increase the accumulation in tumor cells and enhance therapeutic outcomes, liposomes are decorated with surface-attached ligands designed to recognize the specific receptors overexpressed on the surface of various drug-resistant tumor cells. For instance, folate ligands target folate receptor^[82-84]^, transferrin ligands target transferrin receptor^[85,86]^, epidermal growth factor (EGF) protein^[87]^, epidermal growth factor receptor (EGFR) antibody^[88]^, and cetuximab^[89,90]^ target EGFR, and prostate-specific peptide^[91]^ and prostate-specific antigen gene^[92]^ target prostate-specific membrane antigen (PSMA). Additionally, single-stranded DNA aptamer^[93]^ targets forkhead box M1 (FOXM1), biotin^[94-97]^ and fructose^[96]^ target biotin receptor, and humanized anti-human epidermal growth factor receptor 2 (HER2) antibody^[98]^, engineered peptide^[99]^, and bispecific antibody^[100]^ target HER2 receptor. Liposomes can also be designed for controlled release drug delivery to combat cancer drug resistance when exposed to physicochemical or biochemical stimuli, e.g., changes in pH^[82,101,102]^, specific enzymes^[103,104]^, ultrasound^[105,106]^, and so on. Upon exposure to these stimuli, liposomes undergo phase transition, increase membrane permeability, and release loaded active pharmaceutical ingredients at target sites.

**Figure 4:**
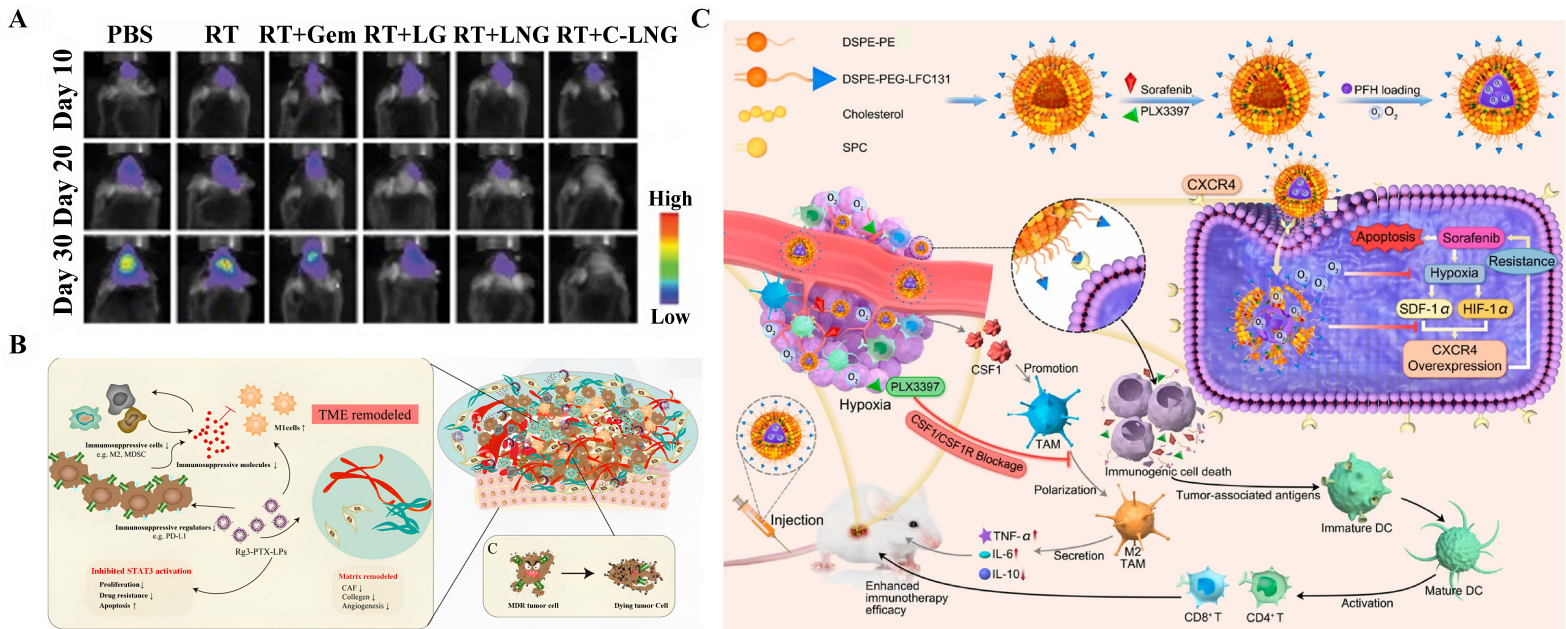
Modulation of liposomal delivery system for overcoming cancer drug resistance. (A) Bioluminescence imaging of brain tissues was conducted at specific time intervals. Kaplan–Meier survival curves were complemented to show the treatment outcomes in mouse models. This figure is quoted with permission from Sun *et al.*^[112]^; (B) Illustration of how ginsenosides Rg3-PTX-LPs reversed cancer drug resistance by remodeling TME and downregulating the expression of P-gp and reduce the efflux of PTX from the cell. This figure is quoted with permission from Zhu *et al.*^[114]^; and (C) Illustration depicting how PFH@LSLP facilitated synergistic antitumor therapy by modulating the hypoxic and immunosuppressive microenvironment for sorafenib-resistant tumor treatment. This figure is quoted with permission from Wang *et al.*^[113]^. RT: Radiotherapy; PFH: perfluorohexane; LSLP: the combination of LFC131 peptides, sorafenib, and PLX3397.

In recent years, liposomes have shown their great potential as a delivery tool for combating cancer resistance, because they play a significant role not only in monotherapy by boosting cellular uptake and drug accumulation^[107]^, but also in combination therapy involving multi-treatments through their co-delivery abilities^[30,103,108-116]^. For example, the furoxans-gemcitabine co-loaded liposome modified with targeted ligands can target the glioblastoma multiforme (GBM) tumor and synergize the radiotherapy (RT) efficacy. Sun et al. developed a reduction-sensitive NO donor conjugate of furoxans-gemcitabine (a RAD51 inhibitor) to serve as a radio-sensitizer for overcoming RT resistance [Figure 4A]^[112]^. The presence of transmembrane efflux pumps is a pivotal challenge for the chemotherapy treatment of multidrug-resistant (MDR) cancers. Previous studies have paid much attention to both monotherapy^[117,118]^ and combination therapy of chemotherapy and transporter inhibitors^[103]^ or siRNA^[102,109,119]^. As the monotherapy, Bai *et al.* developed pemetrexed-loaded D-alpha tocopheryl PEG1000 succinate liposome as a highly appealing strategy to overcome MDR mediated by ABCC5, greatly improving the therapeutic efficacy of pemetrexed in breast cancer^[117]^. A lipidation strategy using phospholipid-conjugation of porphyrin, a photosensitizing drug used in photodynamic treatment, to create benzoporphyrin derivatives has been very informative in evading both P-glycoprotein (P-gp) and ABCG2-mediated transporter in photodynamic treatment-resistant breast cancer. In this study, the authors not only demonstrated the utility of drug-lipid conjugation, but also showed the potential of the lipid material itself to overcome cancer resistance^[118]^. When it comes to combination therapy, Saw *et al.* proposed liposomes conjugated with an extra-domain B-specific aptide to simultaneously deliver MDR1 siRNA and DOX to drug-resistant breast tumors. The silencing of MDR-1 increased the intracellular retention of DOX, leading to appreciable tumor growth inhibition in DOX-resistant MCF7/ADR orthotropic model^[119]^. Furthermore, a TPGS-coated cationic liposome decorated with Bcl-2 siRNA-corona was studied for loading DOX in hepatocellular carcinoma (HCC) for MDR-dual suppression of drug resistance. The system significantly contributed to the inhibition of P-gp efflux and internalization of DOX, thus increasing the *in vivo* antitumor efficacy^[109]^. Similarly, an MDR1-siRNA and DOX co-loaded multifunctional liposome with an antibody-conjugated pH-sensitive system was successfully designed to fight P-gp-related MDR by downregulation of P-gp expression and improving DOX antitumor efficiency^[102]^. Conversely, liposome decorated with PEG and EMC peptide was employed to co-encapsulate DOX and tariquidar, a P-gp inhibitor, to treat triple-negative breast cancer (TNBC). With efficient targeting delivery and a stimuli-responsive system, the liposome has demonstrated superior capability to kill drug-resistant TNBC^[103]^.

To target the tumor sites, specific ligand modification is a promising tool against drug resistance. Ginsenosides Rg3, containing a hydrophilic domain with two glucosyl groups, can be recognized by glucose transporter-1 (GLUT-1) on tumor cells. Substitution of cholesterol with the ginsenosides Rg3, a unique Rg3-based liposome loaded with paclitaxel (PTX), was formulated to target cancer cells and remodel the TME. Rg3 showed great potential to reverse drug resistance not only mediated by proteins including P-gp and programmed cell death ligand-1 (PD-L1), but also mediated by immune cells in TME. Hence, Rg3 itself is expected to endow liposomes with potent capability to target tumor sites, prevent PTX efflux, and increase the damage to MDR cancer cells [Figure 4B]^[114]^. Additionally, a perfluorohexane (PFH)-cored liposome modified with LFC131 peptides, a CXCR4 antagonist, can successfully block the SDF-1α/CXCR4 axis through the reversion of the immunosuppressive microenvironment and restoration of the drug sensitivity of tumor cells, thus re-sensitizing the HCC cells to sorafenib [Figure 4C]^[113]^.

In summary, various liposome-based approaches are carried out for multifunctional liposomes and combination therapy to enhance tumor targeting and drug accumulation, thus overcoming drug resistance in different types of cancer and improving therapeutic outcomes.

### LNPs

The delivery of nucleic acids based on non-viral vectors is a rapidly emerging gene-based therapy that safely reprograms the expression of resistance-associated genes against cancer resistance^[120]^. From the decades of liposomal development, LNPs have emerged within the pharmaceutical field as potentiate cargoes to carry a wide range of therapeutic agents^[69]^. LNPs have stemmed from liposomal technology. It has been developed as a four-component formulation that fundamentally contains phospholipids, cholesterol, ionizable lipids, and PEGylated lipids^[68]^. During the global outbreak of COVID-19, Comirnaty® and Spikevax® are mRNA-encapsulated LNPs for COVID-19 vaccination with FDA approvals in 2020 and 2021, respectively. mRESVIA (mRNA-1345), an mRNA respiratory syncytial virus (RSV) vaccine based on the Spikevax® platform, is also approved by the U.S. FDA in 2024^[121,122]^. LNPs have rapidly drawn robust attention as an effective tool for transporting and protecting genetic materials into cells^[60,123,124]^. Interestingly, as a representative milestone, the RNA-LNPs systems of anticancer drugs were examined and administrated to patients with cancer before the massive attention of the RNA-LNPs-based COVID-19 vaccines^[125]^. Owing to these benefits, the LNPs are further exploited for biomedical applications, particularly for addressing drug resistance issues in cancer therapy. The LNP systems used for delivering anticancer drugs serve to protect and transport genetic materials into cancerous cells, either to erase some reversible modifications or to alter TME^[126]^.

**Figure 5:**
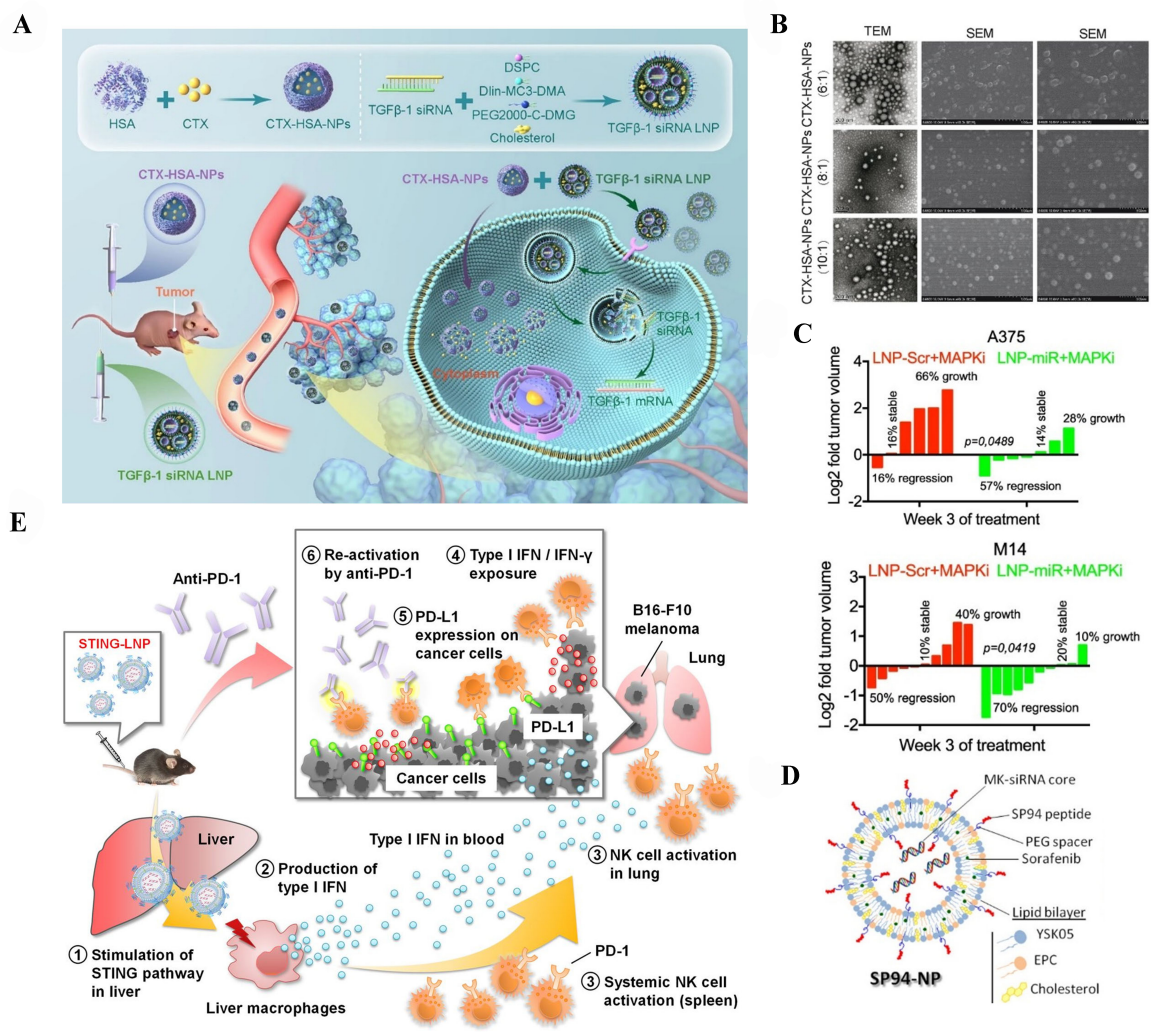
Modulation of LNP carriers for overcoming cancer drug resistance. (A) The use of combination therapy of CTX-HSA-NPs and TGFβ-1 siRNA LNPs in PTX-resistant NSCLC. This figure is quoted with permission from Tan *et al.*^[32]^; (B) The transmission electron microscopy (TEM) and scanning electron microscopy (SEM) images of CTX-HSA-NPs at ratios 6:1, 8:1, and 10:1. This figure is quoted with permission from Tan *et al.*^[32]^; (C) LNP-miRs enhanced the efficacy of MAPKi therapy in xenograft melanoma models. The percentage of tumor reduction was determined relative to the initial volumes in mice injected with A375 or M14 from the groups treated with LNP-Scr + MAPKi or LNP-miRs + MAPKi. This figure is quoted with permission from Fattore *et al.*^[128]^; (D) A versatile lipid-based nanoplatform for precise co-delivery of sorafenib and Midkine siRNA to hepatic cancer cells. This figure is quoted with permission from Younis *et al.*^[33]^; and (E) Overview of mitigating anti-PD-1 resistance through STING-LNP. This figure is quoted with permission from Nakamura *et al.*^[134]^.

For drug resistance in cancer, numerous studies have demonstrated the potential of LNP composition for targeted gene silencing in combination treatment with chemotherapy nanoformulations^[31-33,127-135]^. For example, transforming growth factor beta-1 (TGFβ-1) siRNAs encapsulated in LNPs were used as a combination treatment with cabazitaxel (CTX)-conjugated human serum albumin nanoparticles to treat PTX-resistant non-small cell lung cancer (NSCLC) both *in vivo* and *in vitro*. The cytoplasmic TGFβ-1 mRNA could be silenced by TGFβ-1 siRNA LNPs, thus reversing acquired drug resistance, restoring PTX sensitivity, and synergistically improving anticancer efficacy in PTX-resistant NSCLC [Figures 5A and B]^[32]^. Moreover, LNPs co-encapsulating the selected onco-suppressors miRNAs, miR-199-5p, and miR-204-5p were introduced to evaluate the tumor growth inhibition in combination with targeted therapy. By leveraging the potential antitumor efficacy of mitogen-activated protein kinase inhibitor (MAPKi) therapy and suppressing the development of drug resistance, the miRNA-delivered LNPs could block drug resistance development in BRAF-mutated metastatic melanoma models. These findings strongly suggested the use of RNA-encapsulated LNPs as new combination therapies for metastatic melanoma patients [Figure 5C]^[128]^.

In addition, there is a study about size-controlled LNPs incorporating a highly-selective targeting peptide, a pH-sensitive lipid, and a diversity of phospholipids for selective co-administration of sorafenib and siRNA against the Midkine gene in sorafenib-resistant HCC mice model [Figure 5D]. Results demonstrated that the LNPs showed high tumor accumulation and tissue penetration, and specific gene silencing of Midkine both *in vitro* and *in vivo*^[33,129]^. Another study also showed the potential of targeted LNPs for CRISPR-Cas9 genome editing in two aggressive cancer cell lines. Their LNPs improved the gene editing efficiency of Cas9. Following single local intracerebral administration of this targeted LNPs, Cas9 mRNA and polo-like kinase 1 (PLK1), also known as single-guide RNA (sgRNA), were successfully co-delivered into tumor cells, consequently producing potent tumor growth inhibition and increasing survival in aggressive orthotopic glioblastoma mice^[131]^.

Furthermore, the pH-sensitive LNPs loaded with stimulators of interferon gene (STING) agonists and anti-PD-1 were reported to circumvent the anti-PD-1 resistance in melanoma lung metastasis mouse model. The developed LNPs can protect STING agonists from predegradation and block the PD-1 on NK cells at tumor sites. After entering the cytoplasm, the agonist would stimulate NK cell activation and efficiently induce a synergistic antitumor activity [Figure 5E]^[134]^.

Collectively, the excellent properties of LNPs for protecting and delivering genetic materials into cells endow them with potent capabilities against drug-resistant tumors. They provide efficient platforms for regulating the oncogene expression and improving the efficacy of gene therapy. Importantly, further in-depth explosion is necessary for the safe and efficient clinical application of LNP-based nanomedicines.

### Nanomicelles

Nanomicelles are nanosized colloidal constructs formed by self-assembling of amphiphilic molecules stabilized by emulsifiers with a hydrophobic core and hydrophilic shell in aqueous media. They are usually spherical in shape with a diameter size from 5 to 100 nm. Various oils used as lipid phase in nanomicelles include vegetable oils, glycerides, long-chain unsaturated fatty acids, medium-chain triglycerides, and polyalcohol esters of medium-chain fatty acids. Indeed, different types of emulsifiers, such as phospholipids (e.g., soy lecithin), proteins (e.g., caseinate), surfactants (e.g., Tween 80, sodium dodecyl sulfate), polysaccharides (e.g., modified starch), or polymers [e.g., poly(vinyl alcohol), PEG], are applied to stabilize the interfacial surface to avoid aggregation by steric effects or electrostatic interactions, hydration, and thermal fluctuations^[67]^. Nanomicelles can encapsulate various drug molecules through their delivery systems, such as water-in-oil emulsion (regular micelles), oil-in-water emulsion (reverse micelles), or water-in-oil-in-water emulsion prepared through various techniques, thus increasing the solubility of the drug^[136]^.

Nanomicelles can also be modified with specific ligands or triggered by selective stimuli to improve efficacy and drug release profile^[104,137,138]^. Since 1996, Taxotere®, a docetaxel nanomicelle, is the only FDA-approved nanomicelle formulation launched on the market for locally advanced or metastatic breast cancer after the ineffectiveness of previous chemotherapy [Table 1]. Nowadays, the gradually increasing drug resistance in various types of tumors provides opportunities for nanomicelles to be developed and evaluated in preclinical trials.

**Figure 6:**
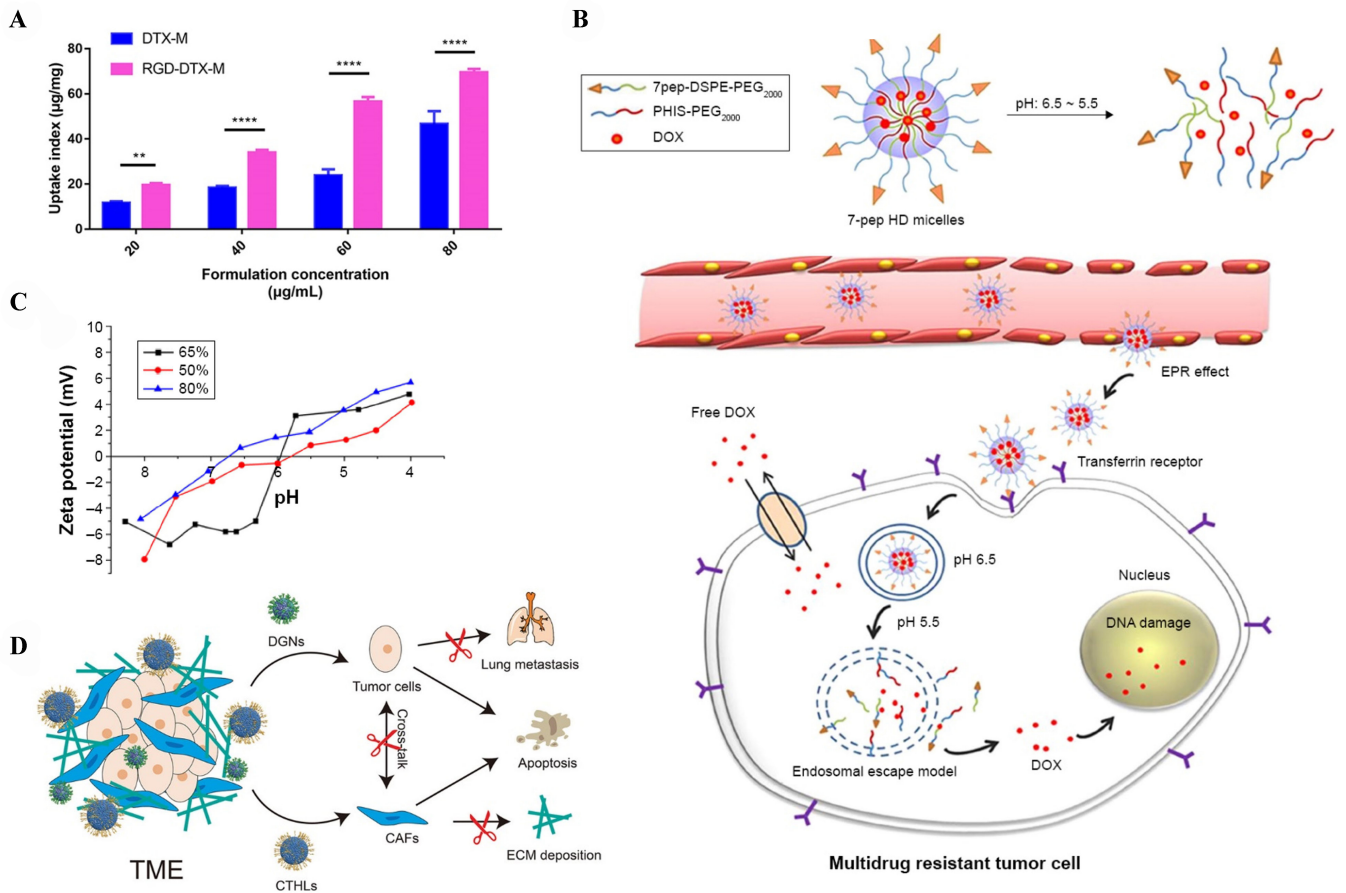
Modulation of lipid nanomicellar delivery system for overcoming cancer drug resistance. (A) *In vitro* cellular uptake of RGD-DTX-M by MDA-MB-231 cells. ^**^P < 0.01; ^****^P < 0.0001. This figure is quoted with permission from Chen *et al.*^[138]^; (B) Diagram depicting the biodegradable pH-sensitive micellar systems and elucidation of the *in vivo* tumor targeting and mechanisms underlying the anti-MDR effect. This figure is quoted with permission from Gao *et al.*^[137]^; (C) The alteration in zeta potential observed in various formulations of pH-responsive PHIS-PEG2000 and DSPE-PEG2000 hybrid micelles. This figure is quoted with permission from Gao *et al.*^[137]^; and (D) Schematic representation of multifaced lipid and micelle formulation employed in HCC. This figure is quoted with permission from Wu *et al.*^[116]^. RGD: (Arg-Gly-Asp) peptides; DTX: docetaxel; MDA-MB-231: human triple negative breast cancer cell line; MDR: multidrug-resistant; HCC: hepatocellular carcinoma.

Targeted nanomicellar systems were developed to challenge drug resistance in cancer. A PEGylated lipid-core nanomicelle decorated with Arg-Gly-Asp peptides (RGD) peptides was engineered to enhance the active targeting of docetaxel (DTX) on TNBC. This delivery system showed high drug loading, long drug retention time in the circulation system, sustained release profile, and reduced side effects of DTX after intravenous administration in MDA-MB-231 xenograft mice model [Figure 6A]^[138]^. Based on lipid-nanomicelles, many combination therapies for cancer resistance have been widely developed^[104,138]^. PEGylated nanomicelles consisting of DSPE-PEG2000 were developed to co-encapsulate PTX and Zos bound with a redox-responsive disulfide linkage. The results suggested that nanomicelles improved intracellular drug accumulation in tumor cells and simultaneously inhibited the MDR tumor growth without systemic toxicity^[104]^.

Similar to liposomes and LNPs, nanomicelles can also be modified with specific receptors or designed to response to stimuli to enhance the therapeutic outcomes^[137]^. For example, as a targeted pH-sensitive nanomicellar system for MDR breast cancer, the DOX-loaded nanomicelle conjugated with PEGylated and transferrin receptor ligand-modified lipid can significantly enhance the cellular uptake and the capacity to damage cancer cells [Figure 6B and C]^[137]^.

Moreover, the combination of lipid nanosystems was also introduced to treat resistant cancer. A multifunctional DOX-loaded nanomicelle, alongside capsaicin and telmisartan co-loaded liposome, was developed to prevent drug resistance development in tumor cells. This combinational therapy can inhibit the cross-talk between tumor cells and cancer-associated fibroblasts in TME, thus reducing the deposition of extracellular matrix and reversing the epithelial-mesenchymal transition (EMT) of tumor cells. The synergistic anti-HCC efficacy with good biocompatibility and safety makes this combinational therapy a potential tool for combating cancer resistance [Figure 6D]^[116]^. These findings provide crucial insights for further strategies in clinical interventions against cancer drug resistance.

## LIPID-BASED NANOMEDICINES FOR DRUG-RESISTANT CANCER THERAPY IN CLINICAL TRIALS

During the last decade, lipid-based nanomedicines have been examined in numerous clinical trials to investigate new standard regimens as monotherapy and combination therapy with other approaches for drug-resistant cancer. Table 3 shows the clinical trials of lipid-based nanomedicines to cope with cancer drug resistance from 2017 to the present.

**Table 3:** Lipid-based nanomedicines for the development of drug-resistant cancer treatments, selected from phase I, II, and III clinical trials

**ClinicalTrials.gov identifiers**	**Sponsor**	**Medication**	**Study title**
**Phase I**			
NCT04718376 (phase I, terminated, 2024)	CSPC ZhongQi Pharmaceutical Technology Co., Ltd.	1. Mitoxantrone hydrochloride liposome	A multicenter, open-label, single-arm, phase Ib study to evaluate the safety and efficacy of mitoxantrone hydrochloride liposome injection in subjects with platinum-resistant or platinum-refractory relapsed ovarian cancer
NCT05483933 (phase I, active, not recruiting, 2024)	Shattuck Labs, Inc.	1. PLD + SL-172154 (SIRPα-Fc-CD40L) 2. Mirvetuximab + SL-172154	An open-label, phase 1b study of SL-172154 (SIRPα-Fc-CD40L) administered with either pegylated liposomal doxorubicin or mirvetuximab soravtansine in subjects with platinum-resistant ovarian cancers
NCT02751918 (phase I, completed, 2019)	Bayer	1. Anetumab ravtansine (BAY94-9343) + PLD	An open-label phase Ib dose escalation study to evaluate the safety, tolerability, pharmacokinetics, immunogenicity, and maximum tolerated dose of anetumab ravtansine in combination with pegylated liposomal doxorubicin 30 mg/m^2^ given every 3 weeks in subjects with mesothelin-expressing platinum-resistant recurrent ovarian, fallopian tube, or primary peritoneal cancer
NCT03591276 (phase I, unknown, 2020)	Shaare Zedek Medical Center	1. Pembrolizumab + PLD	A phase 1b study of combination chemoimmunotherapy with pegylated liposomal doxorubicin (Doxil/Caelyx) and pembrolizumab (Keytruda) in metastatic endocrine-resistant breast cancer
NCT05261490 (phase I, terminated, 2024)	Pfizer	1. Maplirpacept (PF-07901801) + PLD	A phase I/II study of TTI-622 in combination with pegylated liposomal doxorubicin in patients with platinum-resistant ovarian cancer
NCT03639246 (phase I, completed, 2023)	Aravive, Inc.	1. AVB-S6-500 + PLD 2. AVB-S6-500 + PTX 3. Placebo + PLD 4. Placebo + PTX	A phase 1b/2 randomized, controlled study of AVB-S6-500 in combination with PLD or Pac in patients with platinum-resistant recurrent ovarian cancer
NCT03596281 (phase I, active, not recruiting, 2023)	Gustave Roussy, Cancer Campus, Grand Paris	1. Pembrolizumab + PLD 2. Pembrolizumab + bevacizumab	An open-label phase 1 of pembrolizumab in combination with bevacizumab and pegylated liposomal doxorubicin in patients with platinum-resistant epithelial ovarian cancer
NCT03480750 (phase I, completed, 2020)	National Cheng-Kung University Hospital	1. Trientine + PLD + carboplatin	Phase I trial of copper chelator in conjunction with pegylated liposomal doxorubicin and carboplatin in patients with platinum-resistant/refractory epithelial ovarian cancer, tubal cancer, and primary peritoneal cancer
NCT01035658 (phase I, terminated, 2021)	SCRI Development Innovations, LLC	1. Pazopanib + Doxil®	A phase I/II study of the combination of pazopanib and liposomal doxorubicin (Doxil) in patients with advanced relapsed platinum-sensitive or platinum-resistant ovarian, fallopian tube, or primary peritoneal adenocarcinoma
NCT02431559 (phase I, completed, 2022)	Ludwig Institute for Cancer Research	1. Durvalumab + PLD	Phase 1/2 study of chemoimmunotherapy with toll-like receptor 8 agonist motolimod (VTX-2337) + anti-PD-L1 antibody MEDI4736 in subjects with recurrent, platinum-resistant ovarian cancer for whom pegylated liposomal doxorubicin is indicated
NCT04092270 (phase I, recruiting, 2024)	NCI	1. peposertib + PLD	A phase I/Ib dose escalation study of PLD with peposertib (M3814) in platinum-resistant or ineligible ovarian and related cancers with planned expansions in HGSOC and LGSOC
NCT05271318 (phase I, recruiting, 2024)	TILT Biotherapeutics Ltd.	1. TILT-123 + pembrolizumab 2. TILT-123 + pembrolizumab + PLD	A two-part, phase I/Ib, open-label, dose-escalation trial of tumor necrosis factor alpha and interleukin-2 coding oncolytic adenovirus (TILT-123) in combination with pembrolizumab (phase i part) and pembrolizumab and pegylated liposomal doxorubicin (phase ib part) in patients with platinum-resistant or refractory ovarian cancer
**Phase II**			
NCT00913835 (phase II, completed, 2019)	Eli Lilly and Company	1. Olaratumab + PLD 2. PLD or olaratumab monotherapy	Randomized phase 2 trial investigating liposomal doxorubicin with or without anti-PDGFRα monoclonal antibody IMC-3G3 in patients with platinum-refractory or platinum-resistant advanced ovarian cancer
NCT03161132 (phase II, completed, 2023)	Grupo Español de Investigación en Cáncer de Ovario	1. Olaparib 2. PLD	Multicentric single-arm phase II clinical trial to evaluate the safety and efficacy of the combination of olaparib and PLD for platinum-resistant ovarian primary peritoneal carcinoma, and fallopian tube cancer patients
NCT03335241 (phase II, unknown status, 2017)	Sun Yat-sen University	1. Fludarabine + PLD 2. PLD	An open-label, randomized phase II study of fludarabine with pegylated liposomal doxorubicin *vs.* pegylated liposomal doxorubicin alone in patients with platinum-resistant/refractory ovarian cancer
NCT03509246 (phase II, unknown status, 2020)	Seoul National University Hospital	1. PLD + bortezomib	A phase II trial to evaluate the efficacy of bortezomib and pegylated liposomal doxorubicin in patients with BRCA wild-type platinum-resistant recurrent ovarian cancer
NCT04753216 (phase II, completed, 2023)	Northwestern University	1. Bevacizumab + irinotecan liposome	A phase II trial of irinotecan liposome and bevacizumab in women with platinum-resistant ovarian, fallopian tube, or primary peritoneal cancer
NCT03071926 (phase II, unknown status, 2022)	Fudan University	1. PLD	Efficacy and safety of metronomic pegylated liposomal doxorubicin in patients with primary endocrine-resistant advanced breast cancer
NCT02865811 (phase II, completed, 2022)	Dana-Farber Cancer Institute	1. Pembrolizumab + PLD	A phase II study of pembrolizumab combined with PLD for recurrent platinum-resistant ovarian, fallopian tube, or peritoneal cancer
NCT03591276 (phase 1b, unknown status, 2020)	Shaare Zedek Medical Center	1. Pembrolizumab + PLD	A phase 1b study of combination chemoimmunotherapy with pegylated liposomal doxorubicin (Doxil/Caelyx) and pembrolizumab (Keytruda) in metastatic endocrine-resistant breast cancer
NCT05261490 (phase II, terminated, 2024)	Pfizer	1. Maplirpacept (PF-07901801) + PLD	A phase I/II study of TTI-622 in combination with pegylated liposomal doxorubicin in patients with platinum-resistant ovarian cancer
NCT01991210 (phase II, terminated, 2017)	Genentech, Inc.	1. DNIB0600A 2. PLD	A randomized, open-label, multicenter, phase II trial evaluating the safety and activity of DNIB0600A compared to pegylated liposomal doxorubicin administered intravenously to patients with platinum-resistant ovarian cancer
NCT05467670 (phase II, recruiting, 2023)	Haider Mahdi	1. ALX148 + PLD + pembrolizumab	Safety and efficacy of anti-CD47, ALX148 in combination with liposomal doxorubicin and pembrolizumab in patients with recurrent platinum-resistant ovarian cancer: phase II study
NCT03480750 (phase II, completed, 2020)	National Cheng-Kung University Hospital	1. Trientine + PLD + carboplatin	Phase I trial of copper chelator in conjunction with pegylated liposomal doxorubicin and carboplatin in patients with platinum-resistant/refractory epithelial ovarian cancer, tubal cancer, and primary peritoneal cancer
NCT01035658 (phase II, terminated, 2021)	SCRI Development Innovations, LLC	1. Pazopanib + Doxil®	A phase I/II study of the combination of pazopanib and liposomal doxorubicin (Doxil®) in patients with advanced relapsed platinum-sensitive or platinum-resistant ovarian, fallopian tube, or primary peritoneal adenocarcinoma
NCT02431559 (phase II, completed, 2022)	Ludwig Institute for Cancer Research	1. Durvalumab + PLD	A phase 1/2 study of chemoimmunotherapy with toll-like receptor 8 agonist motolimod (VTX-2337) + anti-PD-L1 antibody MEDI4736 in subjects with recurrent, platinum-resistant ovarian cancer, for whom pegylated liposomal doxorubicin is indicated
NCT03268382 (phase II, completed, 2024)	Aprea Therapeutics	1. APR-246 + PLD	PiSARRO-R: p53 suppressor activation in platinum-resistant high-grade serous ovarian cancer, a phase II study of systemic pegylated liposomal doxorubicin chemotherapy with APR-246
NCT06014528 (phase II, recruiting, 2023)	InxMed (Shanghai) Co., Ltd.	1. IN10018 + PLD 2. Placebo + PLD	A multicenter, randomized, double-blind, phase II clinical study of IN10018 in combination with PLD *vs.* placebo in combination with PLD for the treatment of platinum-resistant recurrent ovarian cancer
NCT03804866 (phase II, completed, 2019)	AGC Biologics S.p.A.	1. NGR-hTNF + PLD 2. PLD	NGR018: randomized phase II study of NGR-hTNF plus an anthracycline *vs.* an anthracycline alone in platinum-resistant ovarian cancer
NCT01358071 (phase II, completed, 2018)	AGC Biologics S.p.A.	1. NGR-hTNF + PLD 2. PLD	NGR018: randomized phase II study of NGR-hTNF plus an anthracycline *vs.* an anthracycline alone in platinum-resistant ovarian cancer
NCT02839707 (phase II, active, not recruiting, 2023)	NCI	1. PLD + atezolizumab 2. PLD + bevacizumab + atezolizumab 3. PLD + bevacizumab	A randomized, phase II/III study of pegylated liposomal doxorubicin and CTEP-supplied atezolizumab *vs.* pegylated liposomal doxorubicin, CTEP-supplied bevacizumab and CTEP-supplied atezolizumab *vs.* pegylated liposomal doxorubicin and CTEP-supplied bevacizumab in platinum-resistant ovarian cancer
NCT01593488 (phase II, active, not recruiting, 2023)	NCI, Naples	1. Liposomal cytarabine	Multicentered phase II study evaluating the activity and toxicity of liposomal cytarabine in the treatment of children and adolescents with acute lymphoblastic leukemia with resistant or relapsed central nervous system involvement
NCT00466960 (phase II, completed, 2017)	University of Washington	1. Sargramostim + paclitaxel albumin-stabilized nanoparticle formulation	A phase II trial of GM-CSF with weekly protein-bound paclitaxel (Abraxane™) as chemoimmunotherapy for platinum-refractory/resistant epithelial ovarian, primary peritoneal, and fallopian tube cancer
NCT00499252 (phase II, completed, 2022)	Gynecologic Oncology Group	1. Paclitaxel albumin-stabilized nanoparticle formulation	A phase II evaluation of Abraxane® in the treatment of recurrent or persistent platinum-resistant ovarian, fallopian tube, or primary peritoneal cancer
NCT03531827 (phase II, terminated, 2022)	NCI	1. Enzalutamide + CRLX101	A single-arm phase II study combining CRLX101, a nanoparticle camptothecin, with enzalutamide in patients with progressive metastatic castration-resistant prostate cancer following prior enzalutamide treatment
**Phase III**			
NCT02580058 (phase III, completed, 2023)	Pfizer	1. Avelumab 2. Avelumab + PLD	A phase 3, multicenter, randomized, open-label study of avelumab (MSB0010718C) alone or in combination with pegylated liposomal doxorubicin *vs.* pegylated liposomal doxorubicin alone in patients with platinum-resistant/refractory ovarian cancer
NCT02421588 (phase III, completed, 2020)	PharmaMar	1. Lurbinectedin (PM01183) 2. PLD + topotecan	Phase III randomized clinical trial of lurbinectedin (PM01183) *vs.* pegylated liposomal doxorubicin or topotecan in patients with platinum-resistant ovarian cancer (CORAIL trial)
NCT01170650 (phase III, terminated, 2021)	Endocyte	1. EC145 + PLD (Doxil®/Caelyx®) 2. Placebo + PLD (Doxil®/Caelyx®)	A randomized, double-blind phase 3 trial comparing EC145 and pegylated liposomal doxorubicin (PLD/Doxil®/Caelyx®) in combination *vs.* PLD alone in participants with platinum-resistant ovarian cancer
NCT01281254 (phase III, terminated, 2017)	Amgen	1. AMG386 + PLD 2. Placebo + PLD	A phase 3, randomized, double-blind trial of PLD plus AMG386 or placebo in women with recurrent partially platinum-sensitive or -resistant epithelial ovarian, primary peritoneal, or fallopian tube cancer
NCT00262990 (phase III, completed, 2020)	Novartis Pharmaceuticals	1. Patupilone 2. PLD	A randomized, parallel group, open-label, active controlled, multicenter phase III trial of patupilone (EPO906) *vs.* pegylated liposomal doxorubicin in taxane-/platinum-refractory/resistant patients with recurrent epithelial ovarian, primary fallopian, or primary peritoneal cancer
NCT02839707 (phase III, active, not recruiting, 2023)	NCI	1. PLD + atezolizumab 2. PLD + bevacizumab + atezolizumab 3. PLD + bevacizumab	A randomized, phase II/III study of pegylated liposomal doxorubicin and CTEP-supplied atezolizumab *vs.* pegylated liposomal doxorubicin, CTEP-supplied bevacizumab and CTEP-supplied atezolizumab *vs.* pegylated liposomal doxorubicin and CTEP-supplied bevacizumab in platinum-resistant ovarian cancer
NCT04729387 (phase III, active, not recruiting, 2024)	Novartis Pharmaceuticals	1. Alpelisib + olaparib 2. PTX or PLD	EPIK-O: a phase III, multicenter, randomized (1:1), open-label, active-controlled study to assess the efficacy and safety of alpelisib (BYL719) in combination with olaparib as compared to single-agent cytotoxic chemotherapy, in participants with no germline BRCA mutation detected, platinum-resistant or refractory, high-grade serous ovarian cancer

Data retrieved from https://clinicaltrials.gov/. PLD: Pegylated liposomal doxorubicin; PTX: paclitaxel; Pac: paclitaxel; PD-L1: programmed cell death ligand-1; NCI: National Cancer Institute; HGSOC: high-grade serous ovarian cancer; LGSOC: low-grade serous ovarian cancer; PDGFRα: platelet derived growth factor receptor-alpha; GM-CSF: granulocyte-macrophage colony-stimulating factor; BRCA: BReast CAncer gene; NGR-hTNF: asparagine-glycine-arginine-human tumour necrosis factor; CTEP: Cancer Therapy Evaluation Program; ALX148: a code name of evorpacept; APR-246: a code name of eprenetapopt; AMG386: a code name of trebananib; CRLX101: a name of nanomedicine composed of a camptothecin conjugated to a cyclodextrin-polyethylene glycol co-polymer; SL-172154 (SIRPα-Fc-CD40L): a bi-functional fusion protein consisting of the extracellular domains (ECDs) of human signal-regulatory protein alpha (SIRPalpha; SIRPa; CD172a) and CD40 ligand (CD40L; CD154; TRAP; TNFSF5) linked via a human Fc domain; MEDI4736: durvalumab; TTI-622: a fusion protein consisting of the CD47-binding domain of human SIRPα linked to the Fc region of human IgG4; AVB-S6-500: batiraxcept; APR-246: eprenetapopt or PRIMA-1MET; DNIB0600A: lifastuzumab vedotin or LIFA; IN10018: ifebemtinib; EC145: vintafolide.

## LIMITATIONS OF LIPID-BASED NANOMEDICINES DEVELOPMENT

Advancements in lipid-based nanomedicines have been driven by innovative drug delivery systems, targeted therapies, and related modern technologies^[50,53]^. Although lipid-based nanomedicines show promising therapeutic advantages, the preparation method and manufacturing at a large scale with reproducibility are still challenging, especially for complex lipid nanocarriers^[53]^. The selection of the synthesis method is crucial to determining their therapeutic applications. Different preparation techniques exhibit different drawbacks, which can affect the properties of both nanocarriers and active pharmaceutical ingredients^[61]^. For example, the degradation of drugs during the manufacturing process occurs due to the heat and force from high-pressurized homogenization and ultrasonication. Non-uniformity in size, zeta potential, and PDI is also observed due to inconsistent synthesis processes such as variations in temperature, mixing speed, and extrusion force from batch to batch^[61]^. Numbers of parameters can significantly affect the physicochemical characteristics of nanoparticles. Precise process control of each parameter and condition is necessary to ensure uniformity and minimize the variation among batches^[61]^. Automated machines, such as microfluidic mixer, can be utilized for this purpose^[139]^. Sterilization of finished products is of particular concern, especially during testing in clinical trials^[140]^. Concerns are also raised regarding the toxicity of residual organic solvents in certain preparation methods^[141]^.

Challenges are also presented in the clinical trial process. Pharmacodynamics and pharmacokinetics are influenced by biological environments and remain significant issues for efficacy and safety in both the short-term and long-term outcomes. Specific storage conditions, such as extremely low temperature requirements, contribute to the complexity of logistics and expenses, as well as impacting therapeutic outcomes. The occurrence of chemical degradation, e.g., lipid oxidation and hydrolysis, can affect their shelf life and efficacy. In addition, stringent regulatory requirements for conducting clinical trials and approval processes are complex and costly^[50,53,57,142]^.

## PERSPECTIVES

To overcome the challenges, the research direction should focus on developing more stable and scalable lipid-based nanomedicines. Method validation and quality control are crucial but compromise cost-effective manufacturing. Investigating the use of novel ligands for targeted delivery and exploring the potential of combination therapies could provide new insights. Long-term preclinical trials are also important for understanding the pharmacokinetics and pharmacodynamics of these systems.

Robust studies confirm the significant progress of lipid-based nanomaterials in improving drug delivery, synergizing therapeutic effects, and overcoming drug resistance. Lipid-based nanomaterials can unlock the advanced applications of potent anticancer agents derived from various natural substances by delivering the phytochemicals, which are often restricted to their limited solubility, to the targeted organs^[143]^. Lipid-based nanomaterials enable additional administration routes to increase therapeutic effects and enhance patient compliance, thereby enabling personalized treatment options. Besides investigating novel cytotoxic agents, there is considerable focus on the identification of new indications for existing clinically approved drugs or repurposing them for cancer therapy. Theoretically, repurposed drugs may help shorten the time required for the research and development process and significantly reduce resistance to current therapeutic regimens^[61,144]^. Utilization of lipid-based nanomaterials will make the repurposed drug more efficient and safe. In the meantime, there is an urgent need for a rapid, high-accuracy tool for lipid-based theranostics to address cancer resistance^[145,146]^. NanoBodipy, a nitroreductase-responsive dye, was recently invented as a smart optical nanoprobe for non-invasive and real-time tumor-targeted imaging. This innovation can accumulate in tumors via the EPR effect, which is beneficial for tumor resection surgery guidance as a theranostic agent to achieve complete tumor removal, thereby reducing the prevalence of drug resistance in remaining cancerous cells^[147]^.

Artificial intelligence (AI) technology is increasingly playing important roles in areas such as lipid nanotechnology design and cancer drug resistance modeling, by gathering existing data and creating excellent algorithms^[148]^. EVONANO, an AI platform, has recently been developed as a simulation platform to design nanomedicines according to the optimized treatment parameters for cancer treatment. This *in silico* model simulates virtual tumor growth and tissue-scale dynamics, integrating machine learning to identify the most effective anticancer nanomedicines^[149]^. It could save both time and cost in lipid-based nanomaterials development and significantly advance the field of nanomedicine.

To the best of our knowledge, technological advancements and the integration of modern technology have remarkably revolutionized cancer research, diagnosis, therapy, and cancer management. Their applications have the potential to transform the administration of various therapeutic agents, including gene therapy and vaccines, thereby significantly impacting clinical practices. Addressing these issues requires multidisciplinary collaboration, innovative engineering solutions, comprehensive preclinical and clinical studies, and well-rounded development of regulations.

## CONCLUSION

The development of cancer drug resistance often limits the effectiveness of treatment regimens and leads to relapse, recurrence, and fatality. The development of lipid-based nanomaterials, stemmed from natural vesicles of human body, presents promising avenues for cancer treatment. These nanomaterials, due to their nano-scale size, biocompatibility, flexibility for surface modification, and production techniques, are currently being investigated for the development of new formulations and modifications of existing anticancer agents to offer viable solutions to combat cancer drug resistance. The progress of nanomedicines based on lipid-based nanomaterials is poised to alleviate global burden and improve human health.

To date, robust studies have shown that lipid-based nanotechnology plays a significant role in overcoming drug resistance in cancer treatment. Integrating various anticancer agents and genetic materials into lipid-based nanomaterials can enhance drug tolerability, reduce drug-induced toxicity, reverse some resistant-related mechanisms, and alter the TME. The superiority of these nanomaterials results in the enhancement and synergy of therapeutic effects of anticancer drugs while reducing the occurrence of cancer drug resistance, ultimately improving sensitivity to traditional drugs and survival rates.

In addition to pharmacogenomics, proteomics, and metabolomics, there is considerable potential for leveraging AI technology in computational modeling, mathematical modeling, and machine learning. These avenues hold promise for developing novel tools to predict disease progression and cancer resistance, thereby advancing personalized medicine. To achieve this ambitious goal, it is imperative to gather and analyze the clinical data of patients suffering from resistant cancer using AI algorithms. Predictive models for the most likely outcomes and progressions of cancer could lead to significant improvement in cancer management. However, the implementation of these new methods in clinical care necessitates extensive studies and time investment. Accelerating the translation of these modern technologies to support cancer treatment requires robust collaborations and networking among researchers, clinicians, hospitals, and industry stakeholders, as well as many relative efforts. Interdisciplinary partnerships play a pivotal role in addressing the complex challenges and harnessing the full potential of lipid-based nanomedicines.

## DECLARATIONS

### Copyright

© The Author(s) 2024.
